# Biofilm Producing Clinical *Staphylococcus aureus* Isolates Augmented Prevalence of Antibiotic Resistant Cases in Tertiary Care Hospitals of Nepal

**DOI:** 10.3389/fmicb.2018.02749

**Published:** 2018-11-27

**Authors:** Sarita Manandhar, Anjana Singh, Ajit Varma, Shanti Pandey, Neeraj Shrivastava

**Affiliations:** ^1^Tri-Chandra Multiple College, Tribhuvan University, Kathmandu, Nepal; ^2^Amity Institute of Microbial Technology, Amity University, Noida, India; ^3^Central Department of Microbiology, Tribhuvan University, Kathmandu, Nepal; ^4^The University of Southern Mississippi, Hattiesburg, MS, United States; ^5^Institute of Biotechnology, Zhejiang University, Hangzhou, China

**Keywords:** biofilm, antibiotic resistance, inducible clindamycin resistance, *ica* genes, *Staphylococcus aureus*

## Abstract

S*taphylococcus aureus*, a notorious human pathogen, is a major cause of the community as well as healthcare associated infections. It can cause a diversity of recalcitrant infections mainly due to the acquisition of resistance to multiple drugs, its diverse range of virulence factors, and the ability to produce biofilm in indwelling medical devices. Such biofilm associated chronic infections often lead to increase in morbidity and mortality posing a high socio-economic burden, especially in developing countries. Since biofilm formation and antibiotic resistance function dependent on each other, detection of biofilm expression in clinical isolates would be advantageous in treatment decision. In this premise, we attempt to investigate the biofilm formation and its association with antibiotic resistance in clinical isolates from the patients visiting tertiary health care hospitals in Nepal. Bacterial cells isolated from clinical samples identified as *S. aureus* were examined for *in-vitro* biofilm production using both phenotypic and genotypic assays. The *S. aureus* isolates were also examined for susceptibility patterns of clinically relevant antibiotics as well as inducible clindamycin resistance using standard microbiological techniques and *D*-test, respectively. Among 161 *S. aureus* isolates, 131 (81.4%) were methicillin resistant *S. aureus* (MRSA) and 30 (18.6%) were methicillin sensitive *S. aureus* (MSSA) strains. Although a majority of MRSA strains (69.6%) showed inducible clindamycin resistance, almost all isolates (97% and 94%) were sensitive toward chloramphenicol and tetracycline, respectively. Detection of *in vitro* production of biofilm revealed the association of biofilm with methicillin as well as inducible clindamycin resistance among the clinical *S. aureus* isolates.

## Introduction

*Staphylococcus aureus* is a prominent cause of both community and healthcare associated infections. It can cause minor skin infections to chronic systemic infections which often lead to treatment failures (Lowy, 1998; Fitzpatrick et al., 2005; Dryden, 2009). The ability of *S. aureus* to cause diverse recalcitrant infections is mainly due to the acquisition of resistance to multiple drugs, its diverse range of virulence factors, and its ability to produce biofilm in indwelling medical devices (Lowy, 1998; Chambers and Deleo, 2009; Archer et al., 2011; Figueiredo et al., 2017). Such biofilm associated infections are often chronic and persistent in nature and are the leading cause of morbidity and mortality in healthcare settings (Moormeier et al., 2013). Biofilms are essentially the extracellular polymeric substances (EPS) that provide unique niches to bacterial cells. Low oxygen availability and nutrient deficiency among others are features of biofilm favoring the development of antibiotic tolerant persister cells (Waters et al., 2016). In addition, biofilm also protect the embedded bacterial cells from the host immune cells thus facilitating the survival of pathogens for a prolonged period. The ability of *S. aureus* to form biofilm on biotic and abiotic surfaces is its major virulence property (Donlan and Costerton, 2002; McCann et al., 2008; Namvar et al., 2013). Biofilm’s EPS is regulated by the expression of polysaccharide intercellular adhesion (PIA) proteins. These proteins are encoded by the gene locus *icaADBC* and mediate cell to cell adhesion, thus facilitating biofilm formation. Indeed, *ica* adhesion genes are involved in the pathogenesis progression facilitating the adhesion mechanisms which explain the importance of these genes in *S. aureus* virulence particularly associated with indwelling medical devices (Otto, 2009; McCarthy et al., 2015). Among intercellular adhesion (*ica*) genes, *icaA* and *icaD* have been reported to play a significant role in biofilm formation (Mack et al., 1996; Cramton et al., 1999). Hence, the detection of the *ica* locus along with the phenotypic detection of biofilm is important in *S. aureus* and it would improve the diagnostic decision for treatment of virulent clinical specimens.

With the advancement in the medical field, use of indwelling hospital devices has been prompted as part of treatment for different diseases. However, this also increases the risk of opportunistic bacterial infections such as staphylococcal infections associated with implanted medical devices including catheters, prosthetic devices, endotracheal tubes etc. (Fux et al., 2005; von Eiff et al., 2005; Barrett and Atkins, 2014) Bacteria within the biofilm are not only protected from the host immune systems but also from the antimicrobial agents contributing to treatment failures and recurrent infections (Kiedrowski and Horswill, 2011; Waters et al., 2016). Although many studies have demonstrated the involvement of *S. aureus* biofilm for persistent infections (Fux et al., 2005; Archer et al., 2011; Figueiredo et al., 2017), effective measures to eradicate biofilm harboring bacterial cells *in-vivo* conditions are still poorly identified. This highlights the importance of understanding the mechanism of biofilm formation and its resistance to antimicrobial substances for a successful treatment. In a resource-limited country like Nepal, early detection of biofilm formation in clinical isolates could be essentially an important practice in prevention and management of nosocomial infections. Although previous studies have demonstrated the prevalence of MRSA cases (Mukhiya et al., 2012; Ansari et al., 2014; Bhatta et al., 2016; Belbase et al., 2017), limited knowledge about prevalence of biofilm associated MRSA is available in clinical samples, especially in hospital device associated infections. In recent study, we intended to evaluate the efficacy of both phenotypic and genotypic methods to detect biofilm production (unpublished data) in the clinical samples, which demonstrated TM among all the phenotypic assays performed showed the best correlation with the genotypic method in detecting biofilm formation. In this study, we aim to examine and report the association of biofilm formation with resistance to various clinically relevant drugs as well as inducible clindamycin resistance (ICR) in the clinical *S. aureus* isolates received in two tertiary care hospitals in Nepal.

## Materials and Methods

### Collection and Identification of Isolates

Clinical isolates collected from tissues and medical devices used in patients undergoing treatment in B & B Hospital and Kathmandu Institute of Science and Technology (KIST) Medical Hospital, Nepal were studied. As in the previous study (unpublished data), the clinical samples received in the laboratory including blood, pus, urine, CVC (central venous catheter), tracheostomy tube, and tissues were examined. From all clinical samples processed during study period, 161 isolates were identified as *S. aureus* following standard microbiological procedures (Cheesebrough, 2012). First, the isolates were identified as staphylococcal strain on the basis of colony morphology on Nutrient agar, Blood Agar and Mannitol Salt Agar, Gram’s stain, and different biochemical tests (Bergey and Holt, 1994). The yellow colored, moist, round, glistening opaque colonies with β or weak hemolysis on blood agar that were Gram positive cocci showing typical staphylococcal bunch were subjected to a series of biochemical tests. The isolates exhibiting positive test result to catalase, slide and tube coagulase, methyl red, Voges Proskauer, nitrate reduction, alkaline phosphatase, urease, gelatin hydrolyzing, fermentative, DNase producer, lactose, mannitol, maltose, mannose, sucrose and trehalose fermenting were confirmed as *S.aureus* (Collee, 1999).

### Antimicrobial Susceptibility Test

The antimicrobial susceptibility test (AST) of all isolates was performed by modified Kirby Bauer disk diffusion technique following the guidelines of clinical and laboratory standards institute (CLSI, 2015). We used different antibiotics based on different mode of action and clinical relevance. The antibiotic disks (HiMedia, India) used were penicillin-G (10 units), cefoxitin (30 μg), ciprofloxacin (5 μg), clindamycin (2 μg), chloramphenicol (30 μg), erythromycin (15 μg), gentamicin (10 μg), tetracycline (30 μg) and cotrimoxazole (25 μg). Strains showing resistance to three or more than three different classes of antibiotics were considered multidrug resistant. Cefoxitin disk was used to detect methicillin resistance. *S*. *aureus* ATCC 25923 was used as control strain in each AST assay along with the test strains.

### Screening of Inducible Clindamycin Resistance

**FIGURE 1:**
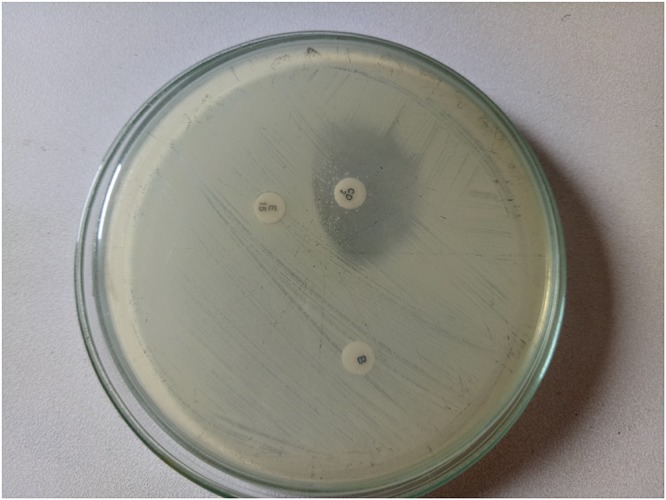
*D*-test showing inducible clindamycin resistance.

The double disk diffusion or D-zone test as outlined in CLSI document M100-S24 (CLSI, 2015) was performed to examine whether the erythromycin resistant isolates expressed inducible clindamycin resistance. Briefly, the bacterial cells from the *S. aureus* isolates were diluted to 0.5 McFarland standard and spread over the Mueller Hinton agar (MHA) plate, on which erythromycin (15 μg) disk and clindamycin (2 μg) disk were placed 15–26 mm edge to edge apart. The plates were incubated at 35°C for 16–18 h in aerobic condition. Flattening of the zone of inhibition of clindamycin adjacent to the erythromycin disk was regarded as *D*-test positive (Figure 1).

### Qualitative Assessment of Biofilm Formation

**FIGURE 2:**
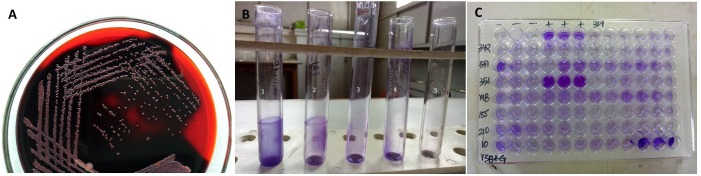
**(A)** Biofilm producing isolates with black colonies on Congo Red Agar medium. **(B)** Biofilm production by tube method. Tube 1:+++, Tube 2:++, Tube 3:+, Tube 4: –, Tube 5: blank. **(C)** Biofilm production by Tissue Culture Plate method.

The biofilm producing strains were screened by qualitative methods such as Congo Red Agar (CRA) method and Tube Method (TM). Strong slime producing reference strain ATCC 35984 (Micobiologics, United States) was used as positive strain in each test performed. Qualitative detection of slime production by CRA method was performed as described by Freeman et al. (1989). Briefly, the Brain Heart Infusion (BHI) Broth (37g/l) supplemented with sucrose (50 g/l), agar (10 g/l) and Congo red dye (0.8 g/l) was used for CRA method. Congo red was prepared as concentrated aqueous solution and autoclaved separately from other constituents and was then added to the mixture when it was cooled to 55°C. The bacterial cells of isolates were streaked on the agar media and incubated aerobically at 37°C for 24 h. The colonies that were black with dark consistency were considered strong slime producers whereas pink colonies were slime non-producers (Figure 2A).

Tube Method was performed as described (Christensen et al., 1985). Briefly, trypticase soy broth (TSB) with 1% glucose were inoculated with the loop-full of inoculum from overnight culture plates and incubated for 24 h at 37°C with shaking at 225 RPM. The tubes were decanted slowly and washed with phosphate buffer saline (PBS, pH 7.3) and air dried. Dried tubes were stained with crystal violet (0.1%) and excess stain was removed and tubes were washed with deionized water. Tubes were then dried in an inverted position and observed for biofilm production. Assays were performed in triplicates three different times. Biofilm was considered positive when a visible film lined the wall and the bottom of tube, and not the ring formation at liquid interface. Based on the intensity of slime production, the result was recorded as strong (+++), moderate (++), weak (+) and none (-) (Figure 2B).

### Quantitative Assessment of Biofilm Formation

The quantitative assay for biofilm formation by TCP method was carried out as described elsewhere (Christensen et al., 1985). Shortly, bacterial cells from fresh culture were inoculated in TSB with 1% glucose and incubated for 24 h at 37°C in stationary condition. After incubation, the culture was diluted (1:100) with fresh TSB medium. From this diluted culture, 200 μl was inoculated onto individual wells of sterile, polystyrene; flat-bottom tissue culture plates (Tarson, India). TSB without cells served as negative control to check sterility and non-specific binding of media. The tissue culture plates were incubated for 24 h at 37°C. After incubation, the content of each well was gently removed by pipetting slowly and tapping the plates. The wells were washed four times with PBS (pH 7.3) to remove free-floating planktonic bacteria and air dried. The wells were then fixed with 2% sodium acetate for 5 min and stained with 1% of crystal violet for 15 min, rinsed thoroughly and repeatedly with deionized water. Optical density (OD) of stained adherent bacteria was determined with a micro ELISA auto reader by taking the absorbance at 570 nm (Christensen et al., 1985). Experiments for each strain were performed in triplicates and repeated three times. Biofilm production was categorized as negative, weak and high depending on the OD values of adherent cells such as OD value < 0.120 as negative, those with OD > 0.120 and < 0.240 were regarded as weak biofilm-producers. An OD value > 0.240 was indicative of high biofilm-producing bacterial strains (Figure 2C).

### Bacterial DNA Extraction and PCR

The genomic DNA from each *S. aureus* isolate was extracted using the DNA extraction Kit (Thermo Fischer), following the manufacturer’s instruction. The sequences of *icaA* and *icaD* (accession number U43366) were taken from the GenBank sequence of the National Center for Biotechnology Information (NCBI) database. Primers specific for *icaA* and *icaD* were designed by the Primer3 program and were purchased from Solis Biodyne, (Denmark). The primer used for the detection of *icaA* was forward 5′-TCTCTTGCAGGAGCAATCAA and reverse 5′-TCAGGCACTAACATCCAGCA primer. The two primers include a 188-bp region. For detection of *icaD*, 5′-ATGGTCAAGCCCAGACAGAG was used as a forward primer and 5′-CGTGTTTTCAACATTTAATGCAA was used as a reverse primer with the product size of 198 bp (Supplementary Figure S1).

### Statistical Analysis

The statistical analysis was performed using SPSS 17.0 (SPSS Inc., Chicago, United States) software. Chi-square test was used to compare between groups of clinical isolates and *P*-values < 0.05 were considered statistically significant.

## Results

### Majority of the Isolates Were MRSA Which Also Showed Higher Rate of Resistance to Other Drugs

Although initially the MRSA infections were confined to the hospitals and patients frequenting to the health care facilities (known as HA-MRSA), community associated MRSA (CA-MRSA) have lately exploded posing economic burden to developed countries (Archer et al., 2011). Given that the prevalence of MRSA cases associated with hospital devices was not reported in Nepal until date, we sought to examine the MRSA strains among device associated staphylococcal isolates. Among 161 *S. aureus* isolates, as indicated by cefoxitin disk diffusion assay, 131 (81.4%) isolates were identified as MRSA and the remaining 30 (18.6%) isolates as MSSA strains. The MRSA isolates were significantly more resistant to majority of the antibiotics than the MSSA strains. However, among all antibiotics, chloramphenicol and tetracycline were found to be the most effective against MRSA strains. AST result showed that 96.9 and 93.9% MRSA isolates were sensitive to chloramphenicol and tetracycline, respectively (Table 1).

**Table 1:** Antimicrobial Susceptibility Test (AST) results of MRSA and MSSA strains.

Antibiotics	Potency (μg/disk)	Resistant cases		
		MRSA (*n* = 131)	MSSA (*n* = 30)	Total (*n* = 161)
Cefoxitin	30	131(100%)	0	131(81.4%)
Penicillin	10	126(96.2%)	27(90%)	158(98.1%)
Tetracycline	30	8(6.1%)	1(3.3%)	9(5.5%)
Clindamycin	2	16(12.2%)	0	16(10%)
Chloramphenicol	30	4(3.1%)	0	4(2.4%)
Ciprofloxacin	5	102(77.9%)	17(56.7%)	119(73.9%)
Erythromycin	15	110(84%)	17(56.7%)	127(78.9%)
Cotrimoxazole	1.25/23.75	72(55%)	18(60%)	90(55.9%)
Gentamicin	10	58(44.3%)	9(30%)	67(41.6%)


*AST of all isolates was performed by modified Kirby Bauer disk diffusion technique following the guidelines of clinical and laboratory standards institute (CLSI, 2015). The number and percentage denotes the prevalence of resistance to the respective antibiotics by respective strains.*

### MRSA Isolates Showed Higher Rate of Inducible Clindamycin Resistance

Clindamycin is frequently used as an alternative treatment to MRSA patients as well as those showing allergies to penicillin. However, *S. aureus* isolates showing *in-vitro* susceptibility to clindamycin can frequently acquire the ICR *in-vivo* leading to treatment failures (Levin et al., 2005). Hence, in this study, we sought to examine the prevalence of ICR among MRSA isolates. Among 161 *S. aureus* isolates, 127 (78.9%) were resistant to erythromycin. When these isolates were subjected to *D*-test, 17 (10.5%) isolates showed resistant to both erythromycin and clindamycin indicating constitutive MLS_B_ phenotype. Out of 145 isolates that were sensitive to clindamycin, 56 (34.8%) also showed positive *D*-test indicating inducible MLS_B_ phenotype, whereas 57 (35.5%) showed true sensitivity to clindamycin as they were *D*-test negative indicating macrolide sensitive (MS) phenotype. The susceptible phenotype (E-S, CD-S) was exhibited by 32 (19.9%) of isolates (Table 2). Among MRSA, the constitutive MLS_B_ and inducible MLS_B_ phenotype was 15 (9.3%) and 42 (26.1%) respectively, while in MSSA, the constitutive MLS_B_ phenotype was 2(1.2%) and inducible MLS_B_ phenotype was 14 (8.7%). When the results were statistically compared, the constitutive MLS_B_ phenotype was determined to be 7.5 times greater (*P* = 0.001, OR 9.9, 95% CI 2.5–39.2) and inducible phenotype 3 times greater (*P* = 0.361, OR 2.4, 95% CI 0.367–15.7) in MRSA than MSSA isolates. Taken together, these results while demonstrating high prevalence of ICR in MRSA cases also indicate the importance of implementation of *D-*test in regular laboratory diagnostics to minimize the risk of treatment failures due to this phenomenon.

**Table 2:** Prevalence of Inducible Clindamycin Resistance (ICR) in MRSA and MSSA strains.

Phenotypes^∗^	MRSA		MSSA		Total	
	(*n*)	%	(*n*)	%	(*n*)	%
E-S, CD-S	20	12.4	12	7.5	32	19.9
E-R, CD-R (constitutive MLSB)	15	9.3	2	1.2	17	10.5
E-R, CD-S (inducible MLSB, *D*-test positive)	42	26.1	14	8.7	56	34.8
E-R, CD-S (MS, *D-*test negative)	54	33.6	3	1.9	57	35.5
Total	131	81.4	30	18.6	161	100


*The double disk diffusion or D-zone test as outlined in CLSI document M100-S24 (CLSI, 2015) was performed to examine whether the erythromycin resistant isolates expressed inducible clindamycin resistance. The flattening of zone of inhibition around the clindamycin placed with erythromycin disk was reported as positive for *D*-test. ^∗^*E* = Erythromycin, CD = Clindamycin, *R* = resistant, *S* = sensitive, MLS_*B*_ = Macrolides, Lincosamides and Streptogramin B.*

### Biofilm Production Was Stronger in MRSA Strains

The structure of biofilm including the robustness and its components show association with antibiotic resistance (Stewart and Costerton, 2001; Ito et al., 2009). We therefore sought to compare the thickness of biofilms formed between MRSA and MSSA strains. Tube method (TM) was used to examine the thickness of biofilm. The result demonstrated that biofilm production was higher in MRSA strains as compared to the MSSA strains not only quantitatively but also qualitatively. Strong biofilm (referring to+++) indicated increased possibility of antibiotic resistance or tolerance that is likely to lead to treatment failures in MRSA infections (Table 3).

**Table 3:** Biofilm production among Staphylococcal isolates by Tube method (TM).

Biofilm production	MRSA	MSSA	Total	*P*-value
Strong (+++)	18 (11.2%)	1 (0.6%)	19 (5.6%)	0.003
Moderate (++)	27 (16.8%)	15 (9.3%)	42 (26.1%)	
Weak (+/-)	86 (53.4%)	14 (46.7%)	100 (62.1%)	


*The visible lining of the inner wall of the tube from overnight culture of the bacterial cells after washing with PBS and stained with crystal violet. Based on the visual intensity of the lining, the tubes were reported as strong, moderate and weak biofilm producers.*

### Biofilm Producing *S. aureus* Strains Reveal Possession of *icaA and icaD* Genes

Intercellular adhesion (*ica*) genes encode PIAs which in turn regulate the biofilm formation (Arciola et al., 2015). Since *icaA* and *icaD* genes are associated with biofilm formation, we sought to examine the possession of these genes in all the isolates studied. The amplification of these genes revealed 45 isolates; 29 MRSA and 16 MSSA strains harboring the *icaA* and *icaD* genes. We observed no significant difference *in-vitro* biofilm production between MRSA and MSSA strains in phenotypic methods while genotypic assay demonstrated MSSA strains possessing significantly higher number of *ica* genes as compared to the MRSA strains (Table 4). This is likely because the MSSA strains are dependent on *icaADBC* encoded PIA to regulate the biofilm formation. On the other hand, MRSA strains more commonly are dependent on extracellular DNA (eDNA), cell surface proteins and major autolysin (McCarthy et al., 2015).

**Table 4:** *In-vitro* detection of biofilm production by MRSA and MSSA strains by commonly used phenotypic assays (CRA, TM, and TCP), and genotypic assay (detection of *icaAD* genes).

Method	Biofilm	MRSA	MSSA	Total	*P*-value
**CRA**	Positive	2(1.2%)	1(0.6%)	3(1.9%)	0.5092
	Negative	129(80.1%)	29(18.0%)	158(98.1%)	
**TM**	Positive	45(28.0%)	16(9.9%)	61(37.9%)	0.0532
	Negative	86(53.4%)	14(8.7%)	100(62.1%)	
**TCP**	Positive	70(43.5%)	14(8.7%)	84(52.2%)	0.5032
	Negative	61(37.9%)	16(9.9%)	77(47.8%)	
**Detection of *ica* genes**	Positive	29(18.0%)	16(9.9%)	45(28%)	0.0006
	Negative	102(63.4%)	14(8.7%)	116(72%)	


### Biofilm Producing Strains That Harbor *icaAD* Genes Were Resistant to More Number of Antibiotics as Compared to the Planktonic Counterparts

Biofilms associated infections are refractory to antibiotics leading to treatment failures and recurrent infections. The embedded bacterial cells are protected from the antibiotics as well as host immune factors by the biofilm matrix facilitating the proliferation of pathogens despite the external stresses (Waters et al., 2016). More importantly, biofilm cells themselves are extremely tolerant to antibiotics leading to recalcitrant and persistent infections (Moormeier et al., 2013). Indeed, as this phenomenon persists, a sub-population eventually develops into the antibiotic resistant clones, causing treatment failures (Fitzpatrick et al., 2005). Indeed, biofilm and antibiotic resistance in *S. aureus* are among the most important virulence factors that function dependent on each other (McCarthy et al., 2015). Hence, in this study, we sought to examine the antibiotic resistance pattern among biofilm forming strains against clinically relevant antibiotics. Our results demonstrate that the rate of resistance to majority of the drugs was higher in biofilm producing strains especially that harbor *icaAD* genes as compared to the *icaAD* negative strains (Table 5).

**Table 5:** Antimicrobial Resistance Pattern and Biofilm Formation in *S. aureus* Isolates.

Antibiotics	Biofilm detection methods		
	TM (*n* = 61)	TCP (*n* = 84)	*icaAD* genes (*n* = 45)
Penicillin	59 (96.7%)	77(91.7%)	43(95.6%)
Cefoxitin	45(73.8%)	46(54.8%)	29(64.4%)
Tetracycline	3(4.9%)	3(3.6%)	3(6.6%)
Clindamycin	2(3.3%)	6(7.1%)	3(6.6%)
Chloramphenicol	2(3.3%)	1(1.2%)	2(4.4%)
Ciprofloxacin	32(52.5%)	70(83.3%)	31(68.9%)
Erythromycin	29(47.5%)	36(42.9%)	28(62.2%)
Cotrimoxazole	20(32.8%)	24(28.6%)	26(57.8%)
Gentamicin	17(27.9%)	20(23.8%)	22(48.9%)


*All the biofilm forming isolates detected by phenotypic and genotypic assays were tested for the antibiotic resistant pattern as described above. The number and percentage denotes the biofilm producing resistant strains against the respective antibiotics.*

## Discussion

Increasing cases of antibiotic resistance in staphylococcal infections pose a serious threat to public health as well as pronounced socio-economic burden across the world. *S. aureus* is a major human pathogen that causes acute to chronic systemic infections which are often refractory to antibiotics leading to treatment failures. Such recalcitrant infections are mainly associated with biofilms formed by *S. aureus* on indwelling devices such as catheters, CVC, prostheses etc. (von Eiff et al., 2005; Murugan et al., 2010; Prasad et al., 2012). Early detection of biofilm forming staphylococci therefore warrants one of the most essential steps for prevention, management, and cure of nosocomial infections. Although the prevalence of MRSA cases in Nepal was previously reported (Mukhiya et al., 2012; Ansari et al., 2014; Bhatta et al., 2016; Belbase et al., 2017), the use of genotypic method to detect biofilm production in MRSA isolates was not published. In this study, we aimed to identify the prevalence of biofilm forming MRSA and MSSA in various clinical samples via different phenotypic and genotypic methods and establish the causal link of biofilm and resistance to multiple drugs in *S. aureus* infections.

In this study, 161 isolates identified as *S. aureus* from different clinical samples were tested for antibiotic susceptibility pattern and *in-vitro* biofilm production by phenotypic and genotypic methods. More than 80% *S. aureus* isolates were found to be methicillin resistant. This result shows higher cases of MRSA in comparison to the previous studies which reported only 19–45.9% of MRSA cases in clinical samples (Mukhiya et al., 2012; Ansari et al., 2014; Bazzoun et al., 2014; Bhatta et al., 2016). In addition, resistance of *S. aureus* to multiple antibiotics such as penicillin, cefoxitin, tetracycline, clindamycin and chloramphenicol was also found to be high. In this study, almost all isolates (98.1%) were resistant to penicillin which was in accordance with previous observations by Ansari et al. (2014) (94.7%) and Belbase et al. (2017) (97.4%). In addition, the rates of resistance to ciprofloxacin (73.9%), erythromycin (78.9%) and cotrimoxazole (55.9%) were also found to be in similar rate to these studies. Ansari et al. (2014) in this study highlighted the risk factors contributing to the increasing rate of resistance in developing countries like Nepal. These factors mainly include lack of regulation of antibiotics availability even without prescription and prescription by unauthorized personnel, self-medication, pharmacies promoting their products through clinicians, and lack of laboratory facilities to detect the antibiotic resistance among others (Ansari et al., 2014). Fortunately, in this study, we observed that a majority of the *S. aureus* including MRSA were susceptible to commonly used antibiotics such as chloramphenicol and tetracycline. Being cheap and easy to administer, these drugs could be established as an ideal option for the preliminary treatment of staphylococcal infections in Nepal.

We detected inducible clindamycin resistance in 80.7% of isolates, which is significantly higher than previous studies that reported only in 12.4 to 22.4% of cases in Nepal (Ansari et al., 2014; Ghasemian et al., 2016; Belbase et al., 2017). In our study, the MS phenotype and constitutive MLS_B_ phenotype was higher among MRSA (33.6 and 26.1%) when compared with MSSA (1.9 and 8.7%). In contrast, previous studies (Schreckenberger et al., 2004; Levin et al., 2005; Sasirekha et al., 2014) showed higher MLS_B_ phenotypes in MSSA as compared to the MRSA strains. The higher incidence of MLS_B_ in our study is explained based on difference in various influencing factors including population studied, the geographical distribution, health care facilities and prevalence of MRSA and MSSA in the particular epidemiological area, (Schreckenberger et al., 2004; Sasirekha et al., 2014). These findings, nonetheless, indicate the importance of *D*-test in routine laboratory diagnostics for preliminary identification of ICR which would be implemented for effective clinical prescription minimizing the treatment failures that are likely to occur due to these phenomena.

Biofilm production was detected by both phenotypic and genotypic assays. On evaluation of phenotypic assays (unpublished data), modified TCP method showed the best correlation with the presence of *icaAD* genes. All the phenotypic methods revealed no significant difference in biofilm production between MRSA and MSSA strains; consistent to the previous study (Ghasemian et al., 2016). However, importantly, strong biofilm formation as measured qualitatively was significantly higher in MRSA strains as compared to the MSSA strains. Due to resource limitation, we could not identify the molecular composition of the biofilms, but, all these results suggest the association between biofilm and antibiotic resistance specially methicillin, inducible clindamycin, ciprofloxacin, erythromycin and cotrimoxazole in clinical isolates in Nepal.

In the previous study, we reported the molecular technique, used for the first time in Nepal, to detect the biofilm formation among *S. aureus* clinical isolates. Amplification of these genes revealed 45 isolates possessing both *icaA* and *icaD* genes including 70% of tube adherent *S. aureus* and all the slime producing strains. We reasoned that the difference in *in-vivo* and *in-vitro* conditions possibly contribute to the physiological changes of the pathogen modulating biofilm formation capabilities. For instance, *ica* genes are expressed in the stressful environment such as high osmolarity, anaerobic condition, high temperature, and sub-inhibitory presence of some antibiotics (Dobinsky et al., 2003; Mirani et al., 2013; Foulston et al., 2014; Moormeier et al., 2014; Arciola et al., 2015). Studies have demonstrated biofilm formation via PIA-independent mechanisms in *S. aureus* (Beenken et al., 2004; Kogan et al., 2006; O’Neill et al., 2007; Rohde et al., 2007). A number of transcriptional regulators have been reported in *ica*-independent biofilm production. These include *araC*- type transcriptional regulator or regulator of biofilm (rbf), which controls the biofilm production by novel regulatory mechanism (Lim et al., 2004). Likewise, biofilm-associated protein (Bap); the first gene is known to form biofilm via *icaADBC* independent in *S. aureus* from bovine mastitis isolates (Cucarella et al., 2001). Although initially, it appeared to be absent in human clinical *S. aureus* isolates, Bap protein has now emerged as being associated with more than 100 surface proteins that are involved in biofilm formation (Lasa and Penades, 2006). In the clinical *S. aureus* isolates of UAMS-1 strain, mutation of *ica* locus showed little effect on biofilm formation, thus, suggesting the presence of additional loci relevant to biofilm formation (Beenken et al., 2004). Also, studies suggest the regulation of biofilm by global regulator *SarA* in *ica*-independent mechanisms (O’Gara, 2007). However, given the undeniable role of *icaADBC* in biofilm matrix formation and that PCR enables rapid diagnosis of slime producing virulent strains assays; implementation of genotypic measure is strongly suggested in routine diagnostic laboratory. Furthermore, *S. aureus* isolates possessing the *icaAD* genes that showed higher resistance rate to number of antibiotics, indicates its importance in routine diagnostics.

## Conclusion

Our findings show that *icaAD* genes are associated with biofilm formation, but absence of these genes may not necessarily exclude this property. Taken together, this study demonstrates the high prevalence of MRSA isolates producing biofilms in clinical staphylococcal samples. Since staphylococcal infections have a significant impact on morbidity and mortality, prevention and management of these infections remain a priority. This study, while bringing additional information about the status of biofilm producing clinical strains and their association with multiple antibiotic resistances, highlights the importance of early detection strategies in routine diagnostics. Implementation of those will help to identify biofilm producing *S. aureus* cases to prevent occurrence of treatment failures of staphylococcal infections in Nepal.

## Author Contributions

SM, NS, and AS designed the study. SM performed the experiments. SM and SP wrote the manuscript. AV edited the manuscript. All authors read and approved the final manuscript.

## Conflict of Interest Statement

The authors declare that the research was conducted in the absence of any commercial or financial relationships that could be construed as a potential conflict of interest.

## Supplementary Material

FIGURE S1 Agarose gel electrophoresis of PCR products.
