# Supplementary figures and images for: Biofilm Producing Clinical Staphylococcus aureus Isolates Augmented Prevalence of Antibiotic Resistant Cases in Tertiary Care Hospitals of Nepal
Source: Front Microbiol. 2018 Nov 27;9:2749. doi: 10.3389/fmicb.2018.02749 (PMC6277500; doi:10.3389/fmicb.2018.02749)

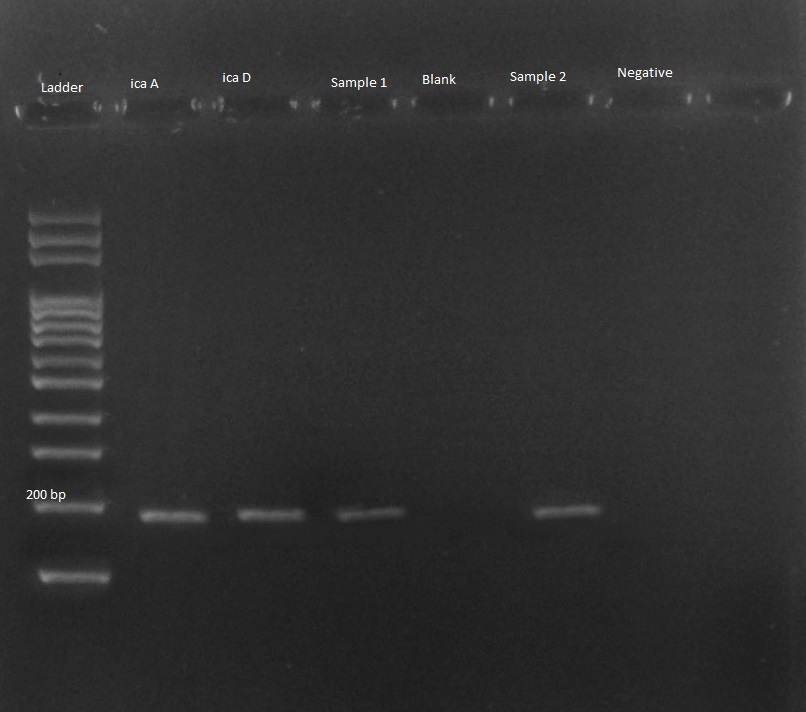

Supplement: FIGURE S1 — Agarose gel electrophoresis of PCR products. [file Image_1.JPEG]
